# Psychotic features, particularly mood incongruence, as a hallmark of severity of bipolar I disorder

**DOI:** 10.1186/s40345-022-00280-6

**Published:** 2022-12-18

**Authors:** Julien Elowe, Julie Vallat, Enrique Castelao, Marie-Pierre F. Strippoli, Mehdi Gholam, Setareh Ranjbar, Jennifer Glaus, Kathleen Merikangas, Benjamin Lavigne, Pierre Marquet, Martin Preisig, Caroline L. Vandeleur

**Affiliations:** 1 Department of Psychiatry, Lausanne University Hospital and University of Lausanne, West Sector, Chemin Oscar Forel 3, Prangins, 1197 Canton of Vaud, Switzerland; 2 Department of Psychiatry, Lausanne University Hospital and University of Lausanne, North Sector, Yverdon, Canton of Vaud, Switzerland; 3 Department of Psychiatry, Psychiatric Epidemiology and Psychopathology Research Center, Lausanne University Hospital and University of Lausanne, Prilly, Switzerland; 4 Child and Adolescent Psychiatry Clinics, University Hospital of Lausanne and University of Lausanne, Lausanne, Switzerland; 5 Genetic Epidemiology Research Branch, Intramural Research Program, National Institute of Mental Health, Bethesda, MD USA; 6 Department of Psychiatry, Center for Psychiatric Neuroscience, Lausanne University Hospital and University of Lausanne, Prilly, Switzerland; 7 International Research Unit in Neurodevelopment and Child Psychiatry, Laval University, Quebec, Canada

**Keywords:** Bipolar I disorder, Psychotic features, Mood congruence, Mood incongruence, Clinical characteristics, Psychiatric comorbidity, Suicide attempts, Treatment

## Abstract

**Background:**

The occurrence of psychotic features within mood episodes in patients with bipolar I disorder (BD I) has been associated in some studies with a more severe clinical and socio-professional profile. In contrast, other studies establishing the associations of psychotic features in BD I, and in particular of mood-congruent (MC) and mood-incongruent (MI) features, with clinical characteristics have yielded contradictory results. However, many pre-existing studies have been affected by serious methodological limitations. Using a sample of thoroughly assessed patients with BD I our aims were to: (1) establish the proportion of those with MI and MC features, and (2) compare BD I patients with and without psychotic features as well as those with MI to those with MC features on a wide array of socio-demographic and clinical characteristics including course, psychiatric comorbidity and treatment.

**Methods:**

A sample of 162 treated patients with BD I (60.5% female, mean age = 41.4 (s.d: 10.2) years) was recruited within a large family study of mood disorders. Clinical, course and treatment characteristics relied on information elicited through direct diagnostic interviews, family history reports and medical records.

**Results:**

(1) A total of 96 patients (59.3%) had experienced psychotic features over their lifetime. Among them, 44.8% revealed MI features at least once in their lives. (2) Patients with psychotic features were much less likely to be professionally active, revealed alcohol abuse more frequently and used health care, particularly inpatient treatment, more frequently than those without psychotic features. Within patients with psychotic symptoms, those with MI features showed more clinical severity in terms of a higher likelihood of reporting hallucinations, suicidal attempts and comorbid cannabis dependence.

**Conclusion:**

Our data provide additional support for both the distinction between BD-I with and without psychotic features as well as the distinction between MI and MC psychotic features. The more severe course of patients with psychotic features, and particularly those with MI psychotic features, highlights the need for thorough psychopathological evaluations to assess the presence of these symptoms to install appropriate treatment.

## Introduction

Clinical studies have estimated the prevalence of psychotic features to be as high as 50–75% in patients with bipolar disorder (BD) (Goodwin and Jamison 1990; Coryell et al. 2001; Azorin et al. 2006; Canuso et al. 2008; Aminoff et al. 2013; Dell'Osso et al. 2017; Keck et al. 2003). A history of psychotic features in BD was found to be made up by 92.5% of delusions and 58% of hallucinations (Bergen et al. 2019). Psychotic symptoms were reported to be more frequent during the manic (Smith et al. 2017) than the depressive episodes of the disorder (Goodwin and Jamison 1990; Canuso et al. 2008; Smith et al. 2017; Prakash et al. 2009; Souery et al. 2011). Despite the frequent occurrence of psychotic symptoms in BD, a recent review revealed that the specific clinical impact of these symptoms is still largely unknown and that the topic has been somewhat neglected in research during more recent years (Smith et al. 2017). Cluster-type analyses have shown mania with psychotic symptoms to be more strongly associated with clinical severity and impairment than non-psychotic mania (Swann et al. 2001; Sato et al. 2002; Haro et al. 2006) and hallucinatory activity was associated with reduced work attainment (Goghari et al. 2013). Previous cross-sectional studies also documented associations between psychotic features with an early onset of the disorder (Bergen et al. 2019; Suominen et al. 2007; Geoffroy et al. 2013; Upthegrove et al. 2015), a longer duration of illness (Coryell et al. 2001), more severe episodes and manic type symptoms (Dell'Osso et al. 2017), worse global functioning scores (Canuso et al. 2008), higher rates of hospitalization (Dell'Osso et al. 2017; Ozyildirim et al. 2010) and a longer duration of hospital stay (Kessing 2004), less insight (Hartung et al. 2017), greater unemployment and lower levels of education, greater family isolation and a higher rate of celibacy (Dell'Osso et al. 2017; Altamura et al. 2019). In contrast to these findings, two earlier studies did not reveal differences between BD with and without psychotic features for a series of demographic, psychosocial, vocational or course variables (Coryell et al. 2001; Keck et al. 2003). Prospective studies have also suggested poor long-term outcomes in patients with BD with psychotic features (Tohen et al. 1990; Tohen et al. 1992a, 1992b; Coryell et al. 1990; Fennig et al. 1996; Carlson et al. 2000; Tsai et al. 2001) although the findings are still mixed (Smith et al. 2017). One study in particular showed that four-year outcomes were generally unfavorable for a first-admission cohort of patients with BD with psychotic features, if patients had had Schneiderian delusions (Carlson et al. 2012).

Regarding the distinction between mood-congruent (MC) and mood-incongruent (MI) psychotic features, the former have been thought to be associated with mood disorders and the latter with schizophrenia, although the DSM-IV proposes that mood incongruence can also occur in manic and depressive episodes of mood disorders (Tohen et al. 1992b). Approximately two-thirds of patients with bipolar I disorder (BD I) with psychosis were found to exhibit the MC subtype (Goodwin and Jamison 1990; Keck et al. 2003; Bergen et al. 2019; Fennig et al. 1996). Generally, diagnostic classification systems assume that the MI subtype is associated with greater severity and a poorer prognosis than the MC subtype (Fennig et al. 1996; Gaudiano et al. 2007). However, research assessing the course of BD in function of the mood congruence of psychotic symptoms is still scarce because most studies did not distinguish between the two forms of psychotic manifestations. A handful of cross-sectional or prospective studies on patients with the MI compared to the MC subtype have distinguished a more severe course in terms of increased number of depressive episodes (Tohen et al. 1992b), a higher lifetime prevalence of anxiety disorders (Keck et al. 2003), a higher number of attempted suicide and hospitalization (Goes et al. 2007), poor lithium responsiveness (Maj et al. 2002) and shorter time in remission, suggesting that the MI subtype may represent a distinct diagnostic entity. However, another study including 238 bipolar patients with psychotic features only found female sex and the risk of comorbid anxiety disorders to be associated with MI (Keck et al. 2003), whereas a second study in the same year found no association between MI psychotic features and syndromal and functional recovery (Tohen et al. 2003). Similarly, a large multicenter study of more than 500 patients with psychotic mania only revealed marginal clinical differences between the MC and MI groups (Azorin et al. 2006). However, MI features were found to occur more frequently in women and tended to be more prevalent in divorced or widowed patients (Azorin et al. 2006).

The inconsistence of results regarding the distinction between BD with and without psychotic features, and of the MI–MC subtypes in particular, is at least partially attributable to small sample sizes, the lack of systematic assessments of the type of psychotic symptoms and methodological heterogeneity across studies, including the diagnostic classification systems used and the ratio of manic and depressive episodes across the course of the disorder. Given that most studies also did not distinguish between MC and MI psychotic features, the association with mood congruence needs to be addressed in large homogeneous samples of thoroughly assessed patients. Hence, our aims were to: (1) establish the proportion of patients with MI and MC features, and (2) compare BD I patients with and without psychotic features, as well as those with MI to those with MC features, on a wide array of socio-demographic and clinical characteristics including course, psychiatric comorbidity and treatment.

## Methods

### Sample

Within a controlled family study of mood disorders (Vandeleur et al. 2014), 162 patients meeting lifetime criteria for BD I with and without psychotic features according to the DSM-IV were consecutively recruited from the inpatient and outpatient facilities of the psychiatric departments of Lausanne and Geneva, in the French-speaking part of Switzerland.

### Procedures

Participants were interviewed by psychologists or psychiatrists who had completed intensive training over a one- to two-month period. Training included supervision of videotaped interviews conducted by clinically experienced senior psychologists.

The collection of information on the patients relied on three sources. First, direct diagnostic information was obtained using the Diagnostic Interview for Genetic Studies (DIGS) (Nurnberger et al. 1994). The French translation of the DIGS (Leboyer et al. 1995) revealed high kappa coefficients for inter-rater reliability and slightly lower coefficients for test–retest reliability for major Axis-I diagnoses including mood and psychotic disorders (Preisig et al. 1999) as well as substance use disorders (SUD) (Berney et al. 2002). The DIGS assesses all symptoms for the last and the most severe manic and for the last and the most severe depressive episodes. The presence and type of psychotic symptoms (hallucinations or delusions) is assessed together with the manic and depressive symptoms. In addition, psychotic symptoms are elicited in a specific psychosis section of the interview. Although, the full list of manic and depressive symptoms are only elicited for the last and the most severe manic and depressive episodes, within a mood summary section the interviewer can document the year of occurrence, the duration, the type of episode (manic, mixed, depressed), the potential manifestation of mood-congruent or mood-incongruent psychotic features as well as inpatient treatment for up to 17 mood episodes. The DIGS was completed by the French version (Leboyer et al. 1991) of the anxiety section of the Schedule for Affective Disorders and Schizophrenia-Lifetime and Anxiety disorders (SADS-LA) (Endicott and Spitzer 1978), which revealed excellent inter-rater and fair-to-good test–retest reliability for the anxiety sections (Leboyer et al. 1991; Rougemont-Buecking et al. 2008). Second, family history information elicited from all participating adult first-degree family members using the Family History-Research Diagnostic Criteria (FH-RDC) was used (Andreasen et al. 1977). The validity of the French version of the FH-RDC has been established through the assessment of agreement between diagnoses derived from direct interviews and family history reports for a series of diagnoses in adults (Rougemont-Buecking et al. 2008; Vandeleur et al. 2008, 2015). Third, clinical records documenting inpatient and outpatient treatment of patients were used.

DSM-IV lifetime diagnoses were assigned according to a best-estimate procedure (Leckman et al. 1982), which relied on the combination of information from the DIGS interview, family history reports as well as medical records. In patients meeting criteria for BD-I with psychotic features, information on the presence and mood congruence of psychotic features were either derived from the DIGS or the medical records. If both MI and MC psychotic features had been reported within one or several mood episodes for a given patient, the most severe type (MI) was assigned for that patient following the DSM-IV. The DIGS also collects information on sociodemographic, clinical and course characteristics of mood disorders as well as the treatment during episodes and across lifespan. Current professional activity was defined as having a professional activity at the time of the completion of the DIGS interview. Socio-economic status (SES) was defined according to the Hollingshead scale (Hollingshead 1975), which takes into account the highest level of education and professional occupation attained by the patient. Clinical and course characteristics also elicited by the DIGS interview included the age at onset of the first mood episode fulfilling DSM-IV criteria for a manic, hypomanic, major depressive or mixed episode, the maximum number of manic symptoms (maximum number of DSM-IV symptoms reported for either the most severe or the most recent manic or mixed episode), the maximum number of depressive symptoms (maximum number of DSM-IV symptoms reported for either the most severe or the most recent major depressive episode), the total number of episodes (sum of manic, mixed and major depressive episodes), intrapersonal mean duration of all, manic or mixed and major depressive episodes, the total time spent in episodes (all, all manic, all mixed and all major depressive episodes), lifetime history of suicide attempts as well as lifetime, current and worst Global Assessment of Functioning (GAF) scores. Potential comorbid psychiatric disorders assessed by the DIGS included generalized anxiety disorder, agoraphobia, panic disorders, social phobia, obsessive compulsive disorder, post-traumatic stress disorder, alcohol abuse or dependence, cannabis abuse or dependence, attention-deficit hyperactivity disorder, childhood separation anxiety disorder, childhood disruptive behavioral disorders (conduct and/or oppositional defiant disorders), anti-social personality disorder, anorexia nervosa, bulimia nervosa and pathological gambling disorder. Consultation of professional healthcare providers as well as the use of any psychotropic drug treatment was assessed for the last and the most severe manic or mixed and depressive episode, whereas inpatient treatment was assessed for each coded mood episode. Moreover, the use of specific psychotropic drugs documented either by the DIGS or by medical records was assessed for lifespan.

### Data analysis

In order to assess the associations of socio-demographic and clinical characteristics with the occurrence of psychotic features in patients with BD I (psychotic features vs. none in the entire sample and mood-incongruent vs. mood-congruent psychotic features among patients with psychotic features), multiple (Montgomery et al. 2013), robust (Huber 2004) or logistic (McCulloch and Searle 2001) regression models were performed, as appropriate. In a first step (Model 1), associations of these characteristics with psychotic features were established using one model for each of these characteristics. These models were adjusted for sex and age. In a second step (Model 2) fully adjusted models were run including sex and age and all variables from Model 1 reaching the lenient *p* < 0.1 level of significance. A first model compared patients with and without psychotic features, a second one compared patients with MI to those with MC psychotic features. All analyses were conducted using the Statistical Analysis System, version 9.4 (SAS Institute, Inc., Cary, NC, USA), and the statistical analyses environment R (R Core Team. R Foundation for Statistical Computing, Vienna, Austria. http://www.R-project.org/).

## Results

### Proportion of mood episode types and sociodemographic characteristics

First, from all analyzed episodes, the mean proportion of depressive episodes was 46.6%, those of manic and mixed episodes were 34.4% and 19.0%, respectively. From a sample of 162 patients (60.5% female, mean age: 41.4, s.d. = 10.2 years), 46.3% were married and the majority (77.2%) were of Swiss origin. A total of 96 out of 162 patients (59.3%) had experienced psychotic features over lifetime, among them 44.8% (n = 43) with MI and 55.2% (n = 53) with MC features. Second, regarding the sociodemographic characteristics of BD-I with versus without psychotic features, Table 1 shows that patients with BD-I with psychotic features were younger and 2.5 times less likely to be professionally active than patients with BD-I without psychotic features. Within patients with psychotic features, the only difference concerned marital status. Those with MI features were more than three times more likely to be married than those with MC features.

**Table 1:** Demographic characteristics by lifetime psychotic feature status

Demographic characteristics	BD I all (n = 162)				BD I with psychotic features (n = 96)			
	Psychotic (n = 96)	Non-psychotic (n = 66)	Test statistic (95% C.I)^a^	*p*	Mood-incongruent (n = 43)	Mood-congruent (n = 53)	Test statistic (95% C.I)^a^	*p*
Women [%]	60.4	60.6	OR = 0.9 (0.5; 1.8)	0.814	60.5	60.4	OR = 1.0 (0.4; 2.3)	0.962
Age [mean (s.d.)]	39.8 (9.9)	43.7 (10.2)	**β = − 3.9** **(− 7.1; − 0.7)**	**0.016**	40.3 (9.8)	39.4 (10.0)	β = 0.9 (**− **3.1; 5.0)	0.646
Swiss nationality [%]	71.9	84.9	*OR* = *0.5* *(0.2; 1.1)*	*0.074*	69.8	73.6	OR = 0.8 (0.3; 2.0)	0.677
Married vs other [%]	39.6	56.1	OR = 0.6 (0.3; 1.2)	0.154	53.5	28.3	**OR = 3.6** **(1.3; 9.8)**	**0.012**
SES ^b^ [mean (s.d.)]	3.3 (1.2)	3.4 (1.1)	*β* = **− ***0.1* *(***− ***0.4; 0.3)*	*0.060*	3.2 (1.1)	3.3 (1.3)	β = **− **0.1 (**− **0.5; 0.4)	0.831
Current professional activity [%]	34.7	56.1	**OR = 0.4** **(0.2; 0.7)**	**0.004**	26.2	41.5	OR = 0.5 (0.2; 1.2)	0.119
Current disability pension [%]	25.0	24.2	OR = 1.2 (0.6; 2.6)	0.629	27.9	22.6	OR = 1.3 (0.5; 3.3)	0.588

Statistically significant differences are indicated in bold. Additional variables reaching the level of p < 0.1 are indicated in italic

*BD I* bipolar I disorder

^a^Models (one model per line) adjusted for sex and age (sex adjusted for age, age adjusted for sex); *p* p-value, *SES* socio-economic status

^b^A value of 3 represents an SES of III (middle class) on the Hollingshead scale

### Clinical and course correlates

BD I patients with and without psychotic features did not differ regarding age at onset of the first episode, although those with psychotic features had a higher maximum number of manic symptoms during the most severe or the most recent manic or mixed episode than those without psychotic features (Table 2). The only other difference involved a lower worst GAF score during lifespan in BD I patients with psychotic features than in patients without psychotic features. Table 2 also shows that, within patients with psychotic features, those with MI features were approximately three times as likely to report any hallucination, auditory hallucinations, somatic hallucinations and visual hallucinations as those with MC features. Patients with MI features were also more than three times more likely to report a suicide attempt and were attributed lower lifetime, current and worst GAF scores than those with MC features.

**Table 2:** Clinical and course characteristics by lifetime psychotic feature status

	BD I all (n = 162)				BD I with psychotic features (n = 96)			
	Psychotic (n = 96)	Non psychotic (n = 66)	Test statistic (95% C.I)^a^	*p*	Mood-incongruent (n = 43)	Mood-congruent (n = 53)	Test statistic (95% C.I)^a^	*p*
Clinical characteristics								
Age of onset of first mood episode [mean (s.d.)]	24.4 (9.5)	27.6 (11.5)	β = − 1.3 (− 4.3; 1.7)	0.388	23.6 (10.0)	25.0 (9.1)	β = − 1.7 (− 5.3; 1.8)	0.328
Maximum number of symptoms during a manic or mixed episode [mean (s.d.)]	*5.5 (1.2)*	*5.0 (1.4)*	***β* = *0.4*** ***(0.0; 0.9)***	**0.041**	*5.3 (1.2)*	*5.7 (1.4)*	*β* = − *0.5* *(*− *1.0; 0.0)*	*0.061*
Maximum number of symptoms during a depressive episode [mean (s.d.)] ^b^	7.1 (1.1)	6.9 (1.2)	β = − 0.0 (− 0.4; 0.4)	0.963	7.1 (1.1)	7.0 (1.1)	β = 0.0 (− 0.4; 0.5)	0.847
Hallucinations or delusions	93.8	–	–	–	97.7	90.6	OR = 4.5 (0.5; 40.2)	0.180
Any Hallucination [%]	52.1	–	–	–	67.4	39.6	**OR = 3.2** **(1.4; 7.7)**	**0.007**
Auditory Hallucinations [%]	43.8	–	–	–	58.1	32.1	**OR = 2.9** ** (1.3; 6.8)**	**0.012**
Somatic Hallucinations [%]	14.6	–	–	–	23.3	7.6	**OR = 3.8** **(1.1; 13.1)**	**0.037**
Olfactory Hallucinations [%]	9.4	–	–	–	16.3	3.8	*OR* = *5.2* *(1.0; 27.3)*	*0.050*
Visual Hallucinations [%]	28.1	–	–	–	41.9	17.0	**OR = 3.5** **(1.4; 9.0)**	**0.009**
Gustative Hallucinations [%]	1.0	–	–	–	2.3	0	–^c^	–^c^
Any Delusion [%]	90.6	–	–	–	93.0	88.7	OR = 1.7 (0.4; 7.4)	0.473
Course characteristics								
Number of episodes								
All [median (IQR)]	5.0 (4.0)	5.0 (2.0)	β = − 0.0 (− 0.2; 0.2)^d^	0.935	6.0 (5.0)	5.0 (3.0)	β = 0.2 (− 0.1; 0.4)^d^	0.218
Manic [median (IQR)]	2.0 (2.0)	1.0 (3.0)	β = 0.1 (− 0.1; 0.3)^d^	0.447	2.0 (4.0)	2.0 (2.0)	β = 0.1 (− 0.2; 0.4)^d^	0.339
Mixed [median (IQR)] ^b^	0.0 (1.0)	0.0 (1.0)	β = 0.0 (− 0.2; 0.3)^d^	0.737	1.0 (1.0)	0.0 (1.0)	*β* = − *0.2* *(*− *0.5; 0.0)*^*d*^	< *0.100*
Depressive [median (IQR)] ^b^	2.0 (3.0)	3.0 (2.0)	β = − 0.2 (− 0.4; 0.4)^d^	0.107	2.0 (3.0)	2.0 (2.0)	β = 0.1 (− 0.2; 0.4)^d^	0.380
Duration of episodes (weeks)								
All [median (IQR)]	13.9 (32.2)	13.9 (21.4)	β = − 0.0 (− 0.4; 0.4)^d^	0.857	14.7 (35.6)	12.5 (26.3)	β = 0.1 (− 0.4; 0.7) ^d^	0.574
Manic [median (IQR)]	5.5 (6.5)	6.3 (6.3)	β = − 0.0 (− 0.3; 0.3)^d^	0.971	5.3 (6.3)	6.0 (5.7)	*β* = − *0.4(*− *0.7; 0.0)*^*d*^	*0.073*
Mixed [median (IQR)]	10.5 (46.3)	12.5 (58.0)	β = 0.0 (− 0.8; 0.8)^d^	0.979	9.0 (72.0)	12.0 (35.0)	β = 0.3 (− 0.7; 1.3)^d^	0.505
Depressive [median (IQR)] ^b^	18.9 (33.5)	16.0 (22.1)	β = 0.2 (− 0.2; 0.6)^d^	0.271	18.9 (33.7)	19.0 (30.3)	β = 0.1 (− 0.5; 0.7)^d^	0.727
Time spent in episodes (weeks)								
All [median (IQR)]	76.0 (135.0)	68.0 (107.0)	β = − 0.2 (− 0.4; 0.4)^d^	0.905	88.0 (156.0)	60.0 (105.0)	β = 0.3 (− 0.3; 0.8) ^d^	0.344
Manic all [median (IQR)]	11.0 (19.0)	14.0 (23.0)	β = − 0.1 (− 0.5; 0.4)^d^	0.801	8.5 (17.0)	13.0 (20.0)	β = − 0.3 (− 0.8; 0.2)^d^	0.289
Mixed all [median (IQR)]	24.5 (89.0)	24.0 (85.0)	β = 0.0 (− 0.9; 0.9)^d^	0.993	24.5 (124.0)	24.0 (73.5)	β = 0.2 (− 0.9; 1.3)^d^	0.743
Depressive all [median (IQR)]^b^	50.0 (92.0)	48.0 (70.0)	β = 0.1 (− 0.3; 0.5)^d^	0.721	52.0 (103.0)	42.5 (66.0)	β = 0.3 (− 0.3; 0.8)^d^	0.335
Suicide attempts [%]	47.4	41.5	OR = 1.3 (0.7; 2.5)	0.460	62.8	34.6	**OR = 3.3** **(1.4; 7.8)**	**0.006**
Lifetime GAF [mean (s.d.)]	60.1 (11.7)	62.3 (11.5)	β = − 1.3 (− 5.0; 2.4)	0.492	55.8 (11.6)	63.6 (10.6)	**β = **− **7.9** **(**− **12.4; **− **3.4)**	**0.001**
Current GAF [mean (s.d.)]	54.9 (15.6)	59.8 (14.5)	*β* = − *4.7* *(*− *9.6; 0.2)*	*0.059*	50.3 (17.1)	58.6 (13.3)	**β = **− **8.4** **(**− **14.6; **− **2.2)**	**0.009**
Worst GAF [mean (s.d.)]	28.3 (9.4)	32.4 (9.2)	**β = − 3.9** **(**− **6.9; **− **0.9)**	**0.011**	24.2 (8.7)	31.7 (8.7)	**β = **− **7.5** **(**− **11.0; **− **3.9)**	** < 0.001**

Statistically significant differences are indicated in bold. Additional variables reaching the level of p < 0.1 are in italic

*BD I* bipolar I disorder, *p* p-value, *IQR* Interquartile range, *GAF* Global Assessment of Functioning score

^a^models adjusted for sex and age (one model per line)

^b^Among patients with a major depressive episode

^c^Odd’s ratio could not be calculated due to only one positive response

^d^Robust regression analysis with log transformation of dependent variable, adjusted for age and sex

### Psychiatric comorbidity

Table 3 shows that the only differences between patients with and without psychotic features were for the lifetime prevalence of agoraphobia and alcohol abuse. Those with psychotic features had a more than three times lower likelihood of reporting agoraphobia but an almost three times higher likelihood of reporting alcohol abuse than those without psychotic features. Within the group of patients with psychotic features, patients from the MI subgroup had a more than four times increased likelihood of cannabis dependence compared to those from the MC group.

**Table 3:** Lifetime psychiatric comorbidity by lifetime psychotic feature status

	BD I all (n = 162)				BD I with psychotic features (n = 96)			
Disorder	Psychotic (n = 96)	Non-psychotic (n = 66)	Test statistic (95% C.I) ^a^	*p*	Mood-incongruent (n = 43)	Mood-congruent (n = 53)	Test statistic (95% C.I) ^a^	*P*
	%	%			%	%		
Generalized anxiety disorder	8.4	13.9	0.5 (0.2; 1.5)	0.235	14.0	3.9	*4.0 (0.8; 21.2)*	< *0.100*
Agoraphobia	5.3	13.9	**0.3 (0.1; 0.9)**	**0.032**	4.7	5.8	0.8 (0.1; 5.0)	0.806
Panic disorders	9.4	6.1	1.5 (0.4; 5.5)	0.500	14.0	5.7	–^b^	–^b^
Social phobia	16.8	21.5	0.7 (0.3; 1.5)	0.357	18.6	15.4	1.3 (0.4; 3.7)	0.811
Obsessive–compulsive disorder	11.6	7.7	1.5 (0.5; 4.5)	0.518	16.3	7.7	2.3 (0.6; 8.6)	0.200
Post-traumatic stress disorder	2.1	3.1	–^b^	–^b^	4.7	0	–^b^	–^b^
Alcohol abuse	21.9	9.2	**2.9 (1.1; 7.8)**	**0.039**	25.6	18.9	1.4 (0.5; 4.0)	0.472
Alcohol dependence	17.7	27.7	0.6 (0.3; 1.2)	0.136	23.3	13.2	2.0 (0.7; 5.8)	0.230
Cannabis abuse	4.2	4.6	0.7 (0.1; 3.2)	0.601	4.7	3.8	1.3 (0.2; 9.8)	0.828
Cannabis dependence	11.5	4.6	2.2 (0.6; 8.4)	0.254	18.6	5.7	**4.5 (1.1; 19.5)**	**0.042**
Attention-deficit hyperactivity disorder	10.5	10.8	0.9 (0.8; 2.5)	0.816	16.3	5.8	3.3 (0.8; 14.2)	0.662
Childhood separation anxiety disorder	12.6	15.4	0.7 (0.3; 1.7)	0.400	16.3	9.6	1.9 (0.5; 6.4)	0.323
Childhood disruptive behavioral disorders ^c^	14.7	6.2	2.5 (0.8; 8.2)	0.132	18.6	11.5	1.8 (0.6; 5.9)	0.317
Anti-social personality disorder	4.2	7.7	0.4 (0.1; 1.7)	0.224	4.7	3.9	1.5 (0.2; 12.1)	0.712
Anorexia nervosa	1.1	3.1	–^b^	–^b^	0	1.9	–^b^	–^b^
Bulimia nervosa	6.3	9.2	0.6 (0.2; 2.0)	0.411	7.0	5.8	–^b^	–^b^
Pathological gambling	4.2	1.5	–^b^	–^b^	7.0	1.9	–^b^	–^b^

Statistically significant differences are indicated in bold. Additional variables reaching the level of p < 0.1 are in italic

*BD I* bipolar I disorder, *p* p-value

^a^Models (one model per line) adjusted for age and sex

^b^Odd’s ratios could not be calculated due to few affected patients

^c^Including oppositional defiant and conduct disorders

### Treatment characteristics

Table 4 reveals that, compared to BD I patients without psychotic features, those with psychotic features were approximately three times more likely to have consulted a professional healthcare provider during their most recent or most severe manic or mixed episode and to have used any psychotropic drug during these episodes. Regarding the lifetime mood-stabilizing treatment, there were no significant differences between patients with psychotic features and those without psychotic features. However, patients with psychotic features had had a higher number of psychiatric hospitalizations than those without. There were no further differences between these two groups regarding depression treatment characteristics. Within the group of psychotic patients, there were no group differences between those with MI and those with MC features on any treatment characteristics.

**Table 4:** Treatment characteristics by lifetime psychotic feature status

	BD I all (n = 162)				BD I with psychotic features (n = 96)			
	Psychotic (n = 96)	Non-psychotic (n = 66)	Test statistic	*p*	Mood-incongruent (n = 43)	Mood-congruent (n = 53)	Test statistic	*p*
			(95% C.I)^a^				(95% C.I)^a^	
Treatment characteristics of most severe or most recent episode								
Consultation of a professional healthcare provider for manic or mixed episode [%]	85.0	66.7	**OR = 2.9 (1.3; 6.5)**	**0.009**	88.4	82.0	OR = 1.7 (0.5; 5.5)	0.387
Any drug treatment for manic or mixed episode [%]	80.4	59.0	**OR = 3.0 (1.4; 6.3)**	** < 0.050**	85.7	76.0	OR = 2.0 (0.7; 6.0)	0.214
Consultation of a professional healthcare provider for depressive episode ^b^ [%]	93.6	96.6	OR = 0.6 (0.1; 3.3)	0.549	97.1	90.9	OR = 3.4 (0.4; 32.3)	0.287
Any drug treatment for depressive episode ^b^ [%]	85.7	86.0	OR = 1.5 (0.5; 4.5)	0.507	93.9	79.6	*OR* = *4.8 (0.8; 27.8)*	*0.080*
Lifetime mood stabilizers								
Lithium only [%]	8.3	20.0	*OR* = *0.4 (0.2; 1.1)*	*0.063*	7.0	9.4	OR = 0.8 (0.2; 4.0)	0.771
Anticonvulsants only [%]	5.2	6.2	OR = 0.7 (0.2; 2.7)	0.575	7.0	3.8	OR = 2.3 (0.3; 15.7)	0.387
Antipsychotics only [%]	18.1	10.0	OR = 2.0 (0.7; 5.5)	0.182	12.2	22.6	OR = 0.5 (0.2; 1.5)	0.196
Combinations								
Lithium and anticonvulsants [%]	1.0	3.1	–^c^	–	0	1.9	–^c^	–
Lithium and antipsychotics [%]	28.1	20.3	OR = 1.6 (0.7; 3.4)	0.244	32.6	24.5	OR = 1.5 (0.6; 3.7)	0.371
Anticonvulsants and antipsychotics [%]	18.8	10.9	OR = 1.6 (0.6; 4.3)	0.310	18.6	18.9	OR = 1.0 (0.4; 3.0)	0.945
Lithium and anticonvulsants and antipsychotics) [%]	14.6	14.1	OR = 1.3 (0.5; 3.3)	0.616	14.0	15.1	OR = 0.9 (0.3; 2.9)	0.870
Number of psychiatric hospitalizations [mean (s.d)]	3.8 (2.7)	2.5 (2.3)	**β = 1.1 (0.3; 1.9)**	**0.006**	3.9 (3.2)	2.9 (2.2)	*β* = *1.0 (− 0.1; 2.0)*	*0.084*

Statistically significant differences are indicated in bold. Additional variables reaching the level of p < 0.1 are in italic

BD I bipolar I disorder, p p-value

^a^Models adjusted for sex and age (one model per line)

^b^Among patients with a major depressive episode

^c^Odd’s ratios could not be calculated due to low numbers

### Fully adjusted models

Table 5 depicts the results of the two fully adjusted models that simultaneously included all variables associated with psychotic features using the lenient statistical significance level of *p* < 0.1 according to the previous models. **Model 1** comparing BD I participants with psychotic features to those without psychotic features only confirmed the associations between psychotic features and younger age, a decreased likelihood of agoraphobia and an increased likelihood of alcohol abuse, whereas professional activity and number of psychiatric hospitalizations did not reach the level of statistical significance. **Model 2** comparing patients with MI to those with MC features only confirmed the association between MI features and lower worst GAF scores, whereas the associations with three forms of hallucinations as well as that with suicidal attempts did not reach the level of statistical significance despite ORs of more than two and four, respectively.

**Table 5:** Associations of demographic, clinical and treatment characteristics with lifetime psychotic feature status according to two fully adjusted models

	Model 1^a^ Psychotic versus Non-psychotic BD I (n = 155)		Model 2^a^ Mood-incongruent versus Mood-congruent BD I (n = 70)	
	OR (95% C.I)^b^	*p*	OR (95% C.I)^b^	*p*
Age	**0.56 (0.4–0.8)**	**0.007**	–	
Swiss nationality	0.45 (0.2; 1.1)	0.119	–	
Married vs other	–		3.55 (0.95; 15.7)	0.073
SES^c^	1.0 (0.7; 1.4)	0.991	–	
Current professional activity	0.48 (0.2; 1.0)	0.064	–	
Number of manic symptoms	1.3 (1.0; 1.8)	0.106	0.77 (0.4–1.4)	0.373
Auditory hallucinations	–		1.38 (0.3; 6.9)	0.695
Somatic hallucinations	–		3.34 (0.3; 43.5)	0.312
Olfactory hallucinations	–		2.03 (0.2; 24.7)	0.537
Visual Hallucinations	–		3.30 (0.8; 17.0)	0.127
Number of mixed episodes	–		0.74 (0.4; 1.4)	0.308
Length of manic episodes	–		0.61 (0.2–1.6)	0.343
Suicide attempts	–		4.1 (1.0; 21.3)	0.072
Lifetime GAF	–		1.24 (0.4; 3.6)	0.687
Current GAF	0.92 (0.6; 1.4)	0.698	0.64 (0.2; 1.7)	0.377
Worst GAF	0.66 (0.4; 1.0)	0.07	**0.41 (0.2; 0.9)**	**0.029**
Generalized anxiety disorder	**–**		1.53 (0.0; 117.9)	0.830
Agoraphobia	**0.17 (0.0; 0.7)**	**0.014**	**–**	
Alcohol abuse	**3.32 (1.1; 11.1)**	**0.047**	–	
Cannabis dependence	–		1.17 (0.1; 13.2)	0.898
Consultation for most severe or most recent manic or mixed episode	0.94 (0.2; 4.4)	0.932	–	
Any medication for most severe or most recent manic or mixed episode	2.56 (0.6; 11.2)	0.196	–	
Any medication for most severe or most recent depressive episode	–		0.58 (0.1–2.6)	0.489
Lifetime lithium monotherapy	0.63 (0.2; 2.1)	0.463		
Number of psychiatric hospitalizations	1.19 (1.0; 1.5)	0.063	1.15 (0.9; 1.6)	0.353

Statistically significant values are indicated in bold

*BD I* bipolar I disorder, *p* p-value, *GAF* Global Assessment of Functioning scores

^a^Only patients with complete data for all assessed variables were kept in the final models

^b^Odd’s ratio and 95% Confidence Intervals, models include variables associated with psychotic feature status according to the previous analyses using the significance level of *p* < 0.1

^c^Socio-economic status according to the Hollingshead scale

## Discussion

Using a consecutively selected sample of patients with BD I that was thoroughly characterized using information from direct semi-structured diagnostic interviews, family history reports and medical records, the main findings of our study were that: (1) more than half of patients with BD I had experienced psychotic features over lifetime with an almost equal distribution of MI and MC features; (2) in BD I patients with psychotic features, we found greater illness severity in terms of lower worst GAF scores and higher healthcare use; and (3) in BD I patients with MI compared to those with MC psychotic features, we also found indicators of greater illness severity in terms of the higher likelihood of the occurrence of hallucinations and the risk of suicidal attempts.

The observed proportion of nearly 60% of patients who had experienced psychotic features over lifetime lies within the range provided by earlier studies of treated samples (50–75%) (Goodwin and Jamison 1990; Coryell et al. 2001; Azorin et al. 2006; Canuso et al. 2008; Aminoff et al. 2013), whereas our proportion of nearly 50% of patients with MI among those with psychotic features was higher than those of other studies, which generally found 20–30% with MI features among patients with psychotic features (Keck et al. 2003; Bergen et al. 2019; Fennig et al. 1996). Our restriction to patients with BD I could in part explain our high proportion of patients with MI features as compared to previous studies that partially also included patients with bipolar II disorders (Keck et al. 2003). In addition, the use of a best-estimate procedure based on three data sources in our study increased the likelihood in our study to identify MI features (Bergen et al. 2019).

Regarding our findings based on separate models for each variable, the observed much lower proportion of professionally active patients among those with psychotic features, which however was not explained by a higher likelihood of having a disability pension, is consistent with data from earlier studies (Altamura et al. 2019; Marneros et al. 2009). However, with respect to clinical manifestations, course characteristics and comorbid disorders, our data provided evidence for rather modest differences between BD-I with and without psychotic features hereby corroborating the findings of earlier studies (Coryell et al. 2001; Keck et al. 2003). Indeed, the established association of psychotic features with the number of manic symptoms only marginally reached the level of statistical significance whereas the very strong association of psychotic features with lower worst GAF scores needs to be considered as rather tautological given that the occurrence of psychotic features is a criterion for the attribution of lower GAF scores. With respect to comorbid disorders, the three times increased likelihood of lifetime prevalence of alcohol abuse in patients with psychotic features contrasted with the three times lower likelihood of agoraphobia in these patients. While alcohol abuse has already been described to be associated with psychotic features in patients with BD (Dell'Osso et al. 2017), the direction of this association still needs to be determined in future prospective research. In contrast, to our knowledge, the association between psychotic features and lower lifetime prevalence of agoraphobia has never been documented before and first needs to be replicated in other studies. One explanation for this association may be that similar to patients with schizophrenia, those with BD with psychotic features could tend to avoid emotional stimuli in public and prefer to be alone through a process of impaired social functioning, as appears to be the case in schizophrenia (Stain et al. 2014; Oorschot et al. 2012). Another hypothesis could be that this might be paralleled to a lack of insight of uncommon situations or potential danger in unfamiliar surroundings following psychotic experiences. As our study is cross-sectional, the direction of the association cannot be determined, this finding therefore requiring investigation in future prospective studies before any definite conclusions can be drawn. In contrast to clinical and course variables that rather modestly differed between patients with and without psychotic features, treatment characteristics more clearly distinguished the two groups, particularly with respect to healthcare use during manic episodes and the need of inpatient treatment. The assessed clinical and course characteristics, which only marginally distinguished the two groups, did not seem to be those that determined the need for treatment or the professional consequences that differentiated the two groups in a more pronounced way. The small differences in clinical manifestations and course features between BD-I with and without psychotic features on one side and the much larger differences in terms of treatment needs between the two forms of BD I on the other side could be one explanation for contradictory results of previous research with several studies that supported significant differences (Altamura et al. 2019; Marneros et al. 2009; Shi and Jiang 2022) and others that did not (Coryell et al. 2001; Keck et al. 2003).

Regarding the comparison between the patients with MI and those with MC features, differences involved marital status, the likelihood of hallucinations, suicidal attempts, cannabis dependence and the GAF scores. Except for the three times higher proportion of married patients among those with MI as compared to those with MC features, which was surprising and not easy to explain, all other assessed characteristics suggested higher severity of illness in patients with MI features with a three times increased likelihood of all forms of hallucinations and lifetime suicide attempts, lower lifetime, worst and current and GAF scores as well as a more than four times higher likelihood to meet lifetime criteria for cannabis dependence. As far as we know, our observation of a higher occurrence of all forms of hallucinations in patients with MI compared to those with MC features has not been documented before, as studies assessing the congruence and type of psychotic symptoms in mood episodes are still scarce (Smith et al. 2017). The higher likelihood of all forms of hallucinations in BD with MI may indicate higher closeness of this subtype of BD to schizoaffective disorder and schizophrenia (Tohen et al. 1992b). Specific associations between cannabis dependence and MI psychotic features have not been reported previously. Again, the direction of this association is elusive. A series of studies have documented associations between high levels of psychoactive cannabis misuse and increased risk of subsequent psychosis in general through a dose–response effect (Robinson et al. 2022; Hirschtritt et al. 2021), whereas the reverse direction has also been documented with cannabis use in the context of a psychotic disorder worsening symptomatology and the clinical prognosis of the disorder (Hirschtritt et al. 2021; Argote et al. 2022). Further studies are necessary to address the nature and clinical consequences of the comorbidity between cannabis use and MI features. Surprisingly, despite differential symptom manifestations and course characteristics between patients with MI and MC psychotic features, treatment variables did not significantly differ between the two groups. One reason was that the sample sizes of the subgroups by mood congruence were relatively small, which only allowed us to detect group differences for dichotomous variables with large effect sizes, whereas effect sizes of ORs of around three (e.g. for depression treatment characteristics) did not reach the level of statistical significance.

The results of our fully adjusted models need to be interpreted with caution as they include variables related to clinical and course characteristics as well as consequences of the specific subtypes of BD. They typically identify the variables with the strongest association with the disorder. For the comparison between BD-I with and without psychotic features, the model essentially confirmed the differential comorbidity patterns. Regarding the difference between patients with MI psychotic features and those with MC psychotic features, the results of the fully adjusted model need to be taken with even more caution given the limited statistical power. Nevertheless, the GAF score during the most severe episode, which was the only variable that remained significantly associated with mood congruence, still confirmed higher clinical severity of the subtype with MI psychotic features. The more severe course of BD with MI than MC psychotic features could partially account for inconsistent findings of previous research if studies included different proportions of patients with MI and MC psychotic features.

Taken together, our results suggest a more severe clinical picture for BD I patients with psychotic features than those without psychotic features, and for BD with MI as compared to BD with MC psychotic features. The adjunction of manic and psychotic components may indeed constitute a specific subtype of BD (Smith et al. 2017), as is also suggested by previous research on its pathophysiological mechanisms and etiology (Goes et al. 2012; Allardyce et al. 2018). However, although the observed higher clinical severity of BD with MI psychotic features provides support to the DSM-IV (and now repeated in the DSM-5) distinction between MI and MC psychotic features, the nosological position of BD with MI psychotic features on the hypothesized spectrum ranging from BD to schizophrenia is still not definitely determined. Indeed, accumulating molecular genetic evidence provides support for the theory that BD I with psychotic features (Lavebratt et al. 2014) and even the MI subtype (Goes et al. 2012) could be a nosological entity separate from BD without psychotic features. Moreover, white matter abnormalities are suggested to be greater in BD patients with psychotic features compared to those without such features (Sarrazin et al. 2014), whereas cortical response abnormalities are suggested to be greater among BD patients without psychotic features (Hamm et al. 2013). Finally, there are arguments for a decrease in the prefrontal cortex-thalamic connectivity and an increase in the somatosensory-thalamic connectivity in both schizophrenia and BD with psychotic features, even at an early stage of psychosis (Sheffield et al. 2020). Recent evidence from our family study that decomposed mood disorders into manic, depressive and psychotic dimensions supported the independent familial aggregation of all three types of symptoms suggesting that BD patients with psychotic features are likely to be affected by two disorders, BD and psychotic disorder, rather than one disorder involving the two (Vandeleur et al. 2014). In order to further explore the nosological place of BD with psychotic features and in particular that of BD with MI psychotic features, studies that compare clinical, biological and treatment characteristics of BD with MI psychotic features to both BD with MC psychotic features and bipolar schizoaffective disorder would provide additional clues to the question of whether BD with MI psychotic features should be considered as a severe form of BD as assumed by DSM-IV and DSM-5, or alternatively as an intermediate diagnostic entity between BD and schizoaffective disorder, or as a subtype of schizoaffective disorder.

The results of this study should be placed into the context of several limitations that have not already been mentioned. First, our sample was collected in inpatient and outpatient facilities of psychiatric university departments which were likely to treat more severely affected patients with BD I, which is reflected by the relatively high proportion of patients with psychotic features. Second, patients were not recruited during their first psychiatric treatment. Accordingly, if no medical records were available on earlier episodes, this information needed to be taken from retrospective data from the diagnostic interview or family history reports. Third, as the coding of the type of delusions and hallucinations, which are complex phenomena, was based either on the interviewer’s ratings of the symptoms reported by the patient or the psychiatrist’s observations, the classification of psychotic features as either MI or MC may still have been subject to error. This might contribute to explaining differences with previous studies. However, the fact that our study used skilled psychologists as interviewers, who systematically probed for specific types of delusions and hallucinations using the DIGS, and that we employed a best-estimate procedure based on both sources of information minimized the risk of this error. It should be noted that mood congruence criteria have been deemed to be ambiguous as, for instance, the content of delusional thoughts often do not concur with the subjective experience of the underlying mood (Smith et al. 2017; Kumazaki 2011). Future studies could further assess the quality of delusions and hallucinations of mood episodes separately (Smith et al. 2017), using both self-report measures of concurrent mood or emotions and objectively-rated measures of delusions by clinicians, for example. A further idea would be to use ecological momentary assessment approaches to be able to match emotional and cognitive phenomena during psychotic experiences. Fourth, as already mentioned, our subsamples of patients with MI and MC features totalizing around 50 patients each were still relatively small for analyses with frequently dichotomized outcomes, increasing the risk of false negative findings, e.g. for comorbid disorders with relatively rare lifetime prevalence such as GAD for which the two groups did not significantly differ despite an OR of 4.0.

Our results also have several clinical implications. First, given the highly increased risk of professional inactivity in patients with psychotic features, therapeutic and rehabilitative efforts that aim at reducing the impact of psychotic symptoms on the patient’s professional activity are advocated. Second, efforts to detect and treat the frequent comorbid alcohol abuse in patients with psychotic features are also advocated as a means of maximizing the clinical course of BD, as outlined by a recent study (Lagerberg et al. 2021). Third, in patients with MI psychotic features, the largely increased risk of suicidal attempts needs particular clinical attention. Furthermore, both appropriate antipsychotic treatment to contain the increased hallucinatory activity and therapeutic strategies targeting at reducing cannabis dependence are likely to contribute to improve the particularly severe course in these patients.

In conclusion, our data provide additional clinical evidence supporting both the distinction between BD-I with and without psychotic features as well as for the distinction between MI and MC psychotic features. These findings are compatible with those of studies on the biological correlates of the psychotic feature subtypes of BD that show increased severity of BD in the presence of psychosis (Goes et al. 2012; Hope et al. 2013; Tighe et al. 2012). The more severe course of patients with psychotic features and particularly those with MI psychotic features highlights the need for thorough psychopathological evaluations to assess the presence of these symptoms to install appropriate treatment.

## Data Availability

The data used in this article cannot be fully shared as they contain potentially sensitive personal information on participants. The datasets used and/or analyzed during the current study will be made available to individual authors on reasonable request.

## Abbreviations

BD: Bipolar disorder
BD I: Bipolar I disorder
MI: Mood-incongruent psychotic features
MC: Mood-congruent psychotic features
